# Dynamics of multidrug-resistant avian pathogenic *E. coli* biofilm formation on various surfaces and its dispersion with phage antibiotic synergism

**DOI:** 10.1186/s12866-026-05018-3

**Published:** 2026-04-09

**Authors:** Pankhudi Bhutada, Nidhip Joshi, Sunil D. Saroj, Santosh Koratkar

**Affiliations:** Symbiosis School of Biological Sciences, Symbiosis International (Deemed) University, Lavale, Pune, India

**Keywords:** Polypropylene, Stainless steel, Polyvinyl chloride, MDR-APEC, Bacteriophages, Phage-antibiotic synergism, Phage-antibiotic antagonism

## Abstract

Avian pathogenic *Escherichia coli* (APEC) infections are persistent threats to the poultry industry, causing substantial economic losses. This study evaluated the biofilm-forming potential of 32 multidrug-resistant APEC (MDR-APEC) isolates on three common poultry industry surfaces: polypropylene (PP), stainless steel (SS), and polyvinyl chloride (PVC). The results indicated that PVC had the highest biofilm formation rate (82%), followed by SS (79%) and PP (64%). Three isolates that showed moderate to strong biofilm formation across all surfaces were tested for minimum inhibitory and bactericidal concentrations (MICs and MBCs) of Gentamicin (GEN) and Ciprofloxacin (CIP) ranging from 2500 µg/ mL to 31.25 µg/ mL. These isolates exhibited resistance, requiring varying doses for a bactericidal effect. Sensitivity to the phages EscoΦA-06, EscoΦA-07, and EscoΦB-01 was confirmed by bacterial lysis assay. Bacterial killing assays revealed that a multiplicity of infection (MOI) of 100 was optimal for antimicrobial activity. Phage‒antibiotic synergy (PAS) tests revealed effective combinations for dispersing biofilms on all surfaces. Although PAS was predominant, some phage‒antibiotic antagonism (PAA) was observed with Ciprofloxacin combinations. Surface-specific analysis revealed that PP enabled the most efficient biofilm dispersal, whereas PVC proved challenging due to strong biofilm formation. This study offers critical insights into optimizing phage‒antibiotic combinations to combat biofilm-associated challenges in controlling MDR‒APECs infections in poultry. Understanding the synergistic and antagonistic interactions between phages, antibiotics, and surface materials can offer a promising avenue for developing effective antimicrobial strategies for poultry management.

**Graphical Abstract:**

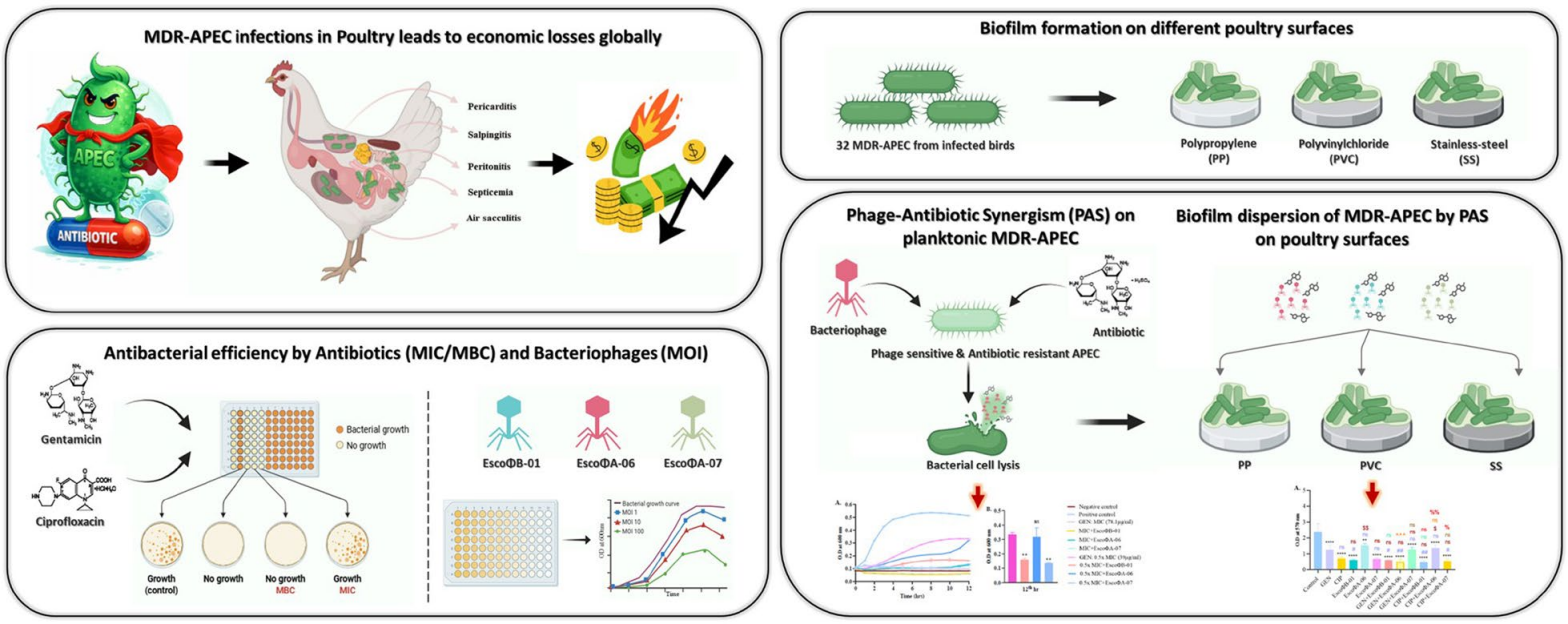

## Introduction

Avian colibacillosis is an infection caused by pathogenic strains of *Escherichia coli* in poultry, turkeys, and other birds [1]. This disease is associated with a range of extraintestinal inflammatory conditions, such as acute fatal septicemia, air sacculitis, chronic respiratory diseases, cellulitis, pericarditis, peritonitis, and salpingitis [2, 3]. Outbreaks of Avian Colibacillosis in poultry farms result in significant economic losses due to carcass condemnation, reduced egg production, morbidity, and mortality [4]. Additionally, the cost of disinfecting poultry spaces and treating infected birds contributes to the economic burden [5].

Colibacillosis in broilers typically starts as a respiratory tract infection when birds inhale fecal dust contaminated with APEC and ammonia [6]. The spread of bacteria may result in various inflammatory conditions, including air-sacculitis and pericarditis, affecting the overall health and productivity of the flock. APEC strains represent a large portion of *E. coli* types and are classified under extraintestinal pathogenic *E. coli* (ExPEC) together with uropathogenic *E. coli* (UPEC), sepsis-associated *E. coli* (SEPEC), and neonatal meningitis *E. coli* (NMEC). Several studies have indicated that APEC and human ExPEC strains share similar virulence-associated genes, suggesting the potential for zoonotic transmission [2, 7].

Antimicrobial therapy has been the primary method for treating APEC infections in the poultry industry [8]. However, the overuse of antimicrobials has led to the development of antimicrobial resistance (AMR) among bacterial pathogens, significantly contributing to the AMR crisis in, the One Health framework impacting human and animal health [9]. However, the AMR issue in *E. coli* poses a serious health hazard and needs to be addressed. Antimicrobial-resistant strains evolve and often interact with the host’s gut microflora, facilitating the spread of resistance genes via mobile genetic elements. Resistance development has also increased due to the ability of bacteria to adhere the surfaces and form biofilms, which are even more difficult to remove with the use of antibiotics. These strains of *E. coli*, particularly those producing extended-spectrum β-lactamases (ESBLs), are resistant to penicillin, tetracycline and cephalosporin, leading to global dissemination [10, 11]. Compared with non-ESBL infections, infections caused by ESBL *E. coli* are responsible for higher economic costs, longer hospital stays, and increased mortality. The use of antimicrobials as growth promoters has been banned in many countries to curb this issue.

Chickens often come into contact with different surfaces in the restricted environment of poultry houses. Stainless steel (SS) is widely used in cages where these birds are housed [12]. Polyvinyl chloride (PVC) pipes are often used to supply water [13], whereas polypropylene (PP) is an essential component of chicken feeders that hold poultry feed [14]. In the case of APEC infection, these materials become potential sites for bacterial adhesion, leading to biofilm formation on these surfaces [15]. Biofilms, or microbial communities, provide an environment for transmitting antibiotic resistance and virulence genes across bacterial strains [16].

APEC strains exhibit a remarkable ability to form stable biofilms, a critical factor that increases their persistence and survival on poultry farms [17]. Biofilms are a physiological state in which bacterial cells adhere to surfaces and are encased in a self-secreted extracellular polymeric matrix composed of proteins, polysaccharides, and nucleic acids [18]. This biofilm growth provides a survival advantage, including facilitating horizontal gene transfer of AMR and virulence-associated genes between bacteria of the same or different species [19]. Therefore, poultry farmers must regularly monitor and clean equipment to prevent the formation of biofilms and reduce the spread of bacterial infections.

Despite the rise in AMR, bacteriophage therapy offers a promising alternative for treating APEC infections. Bacteriophages (phages), which are obligate parasites of bacteria, have emerged as promising candidates for controlling such pathogens [20]. Phages are responsible for inhibiting biofilm formation and the dispersion of mature biofilms [21]. However, phages and their interactions with bacteria need to be studied in detail, including the effectiveness of phages, phage specificity, spectrum of phage activity, strong lytic potential, lysogenic and AMR genes and lack of a regulatory framework [22]. A recent study by Jaroni et al. assessed the efficacy of phage cocktails in eradicating Shigatoxigenic *E. coli* (STEC) on different surfaces, such as polystyrene well plates, SS, and high-density polyethylene [23]. After 16 h of treatment, the STEC populations were reduced to undetectable levels. Similarly, Brás et al. investigated the impact of phage phT4A on *E. coli* biofilm formation on plastic surfaces and reported the highest inhibition rate within 6 h of application [24]. In this context, our study aimed to evaluate the combined effect of phage‒antibiotic synergism on controlling APEC infection and APEC biofilms on three different surfaces, namely, PP, SS and PVC.

## Materials and methods

### Bacterial strains and bacteriophages

Samples were collected from various poultry farms of India and *E. coli* were isolated and confirmed at Symbiosis School of Biological Sciences, Pune, India. Wastewater samples collected and phage isolates EscoΦB-01, EscoΦA-06 and EscoΦA-07 were isolated against the standard *E. coli* strain *E. coli* 8739 [25]. The phages EscoΦB-01 and EscoΦA-07 belongs to the Dhillonvirus family, whereas EscoΦA-06 belongs to the Straboviridae family. The whole genome sequencing and analysis performed earlier by Koratkar et al., 2024, revealed that these phages are free from antibiotic resistance genes, virulence genes, and lysogeny modules. The phage and bacterial isolates were preserved in glycerol at -80 °C until further experiments. All isolated bacterial strains were cultured on 1.5% nutrient agar. They were also cultured on MacConkey’s Agar and Eosin Methylene Blue Agar (EMB). The phages were grown using the agar overlay method, with *E. coli* 8739 as a host [25].

### Procuring polypropylene, stainless steel, and PVC Discs

A 24-well plate (Tarsons Products Ltd.) material made of polypropylene, SS, and PVC coupons was procured from a local hardware store for the study. The discs were 15 mm in diameter and fitted perfectly into the well. The materials had a uniform, smooth surface with no surface irregularities (roughness). The discs were autoclaved at 121 °C and 15 psi before use in all experiments.

### Biofilm formation assay of *E. coli* isolates on different surfaces

The biofilm formation assay was adapted from Necel et al. [26] with minor modifications. Bacterial suspensions were prepared by growing the isolates on 1.5% nutrient agar slants at 37 °C for 24 h, and the optical density was set to 0.3 at 600 nm via a Multiskan SkyHigh Microplate Spectrophotometer (Thermo Scientific™). The sterilised SS and PVC discs were placed in a 24-well plate, with 1 ml of bacterial culture added, and incubated at 37 °C for 16–18 h. After incubation, the biofilms were stained with 0.1% crystal violet, washed with 1x PBS, and destained with 30% glacial acetic acid. The absorbance was recorded at 570 nm.

### Minimum inhibitory concentration (MIC) and minimum bactericidal concentration (MBC)

The MICs of gentamicin (GEN) and ciprofloxacin (CIP) (HiMedia Laboratories, India) were determined by serial dilutions from 2500 µg/mL to 2.5 µg/mL in sterile NB in a 96-well plate [27]. APEC isolates, grown to mid-log phase (O.D. 0.1 600 nm), were exposed to various antibiotic concentrations for 15 h. The growth of the isolates was monitored via a Multiskan SkyHigh Microplate Spectrophotometer (Thermo Scientific™). Bacterial growth of bacterial isolates without antibiotics was termed the positive control in this study. Wells with no visible turbidity were plated on 1.5% nutrient agar and incubated at 37 °C for 24 h to determine the minimum bactericidal concentration (MBC).

### Bacterial killing assay

The in vitro lysis assay was performed as described previously Zhou et al., [28] with a few modifications. Various dilutions of phage titers were prepared. APEC isolates were grown in the mid-log phase, and the O.D. was set to 0.1 at 600 nm. 100 µL of bacteria and 100 µL of phage suspension were mixed such that the MOIs were 1, 10, and 100. Nutrient broth (NB) was used as a negative control, and a bacterial suspension was used as a positive control. For the kinetic assay, the plate was incubated in a Multiskan SkyHigh Microplate Spectrophotometer (Thermo Scientific™) for 12 h. The percent lysis of phage at 12 h was calculated using the following formula.

$$\:Percent\:lysis=\frac{\left(O.D.\:of\:positive\:control-O.D.\:of\:test\:sample\:\right)}{O.D.\:of\:positive\:control}\:x\:100$$

### Phage‒antibiotic synergism assay

In a 96-well plate, antibiotics at the MIC and sub-MIC concentrations were added, followed by phage to obtain 100 MOI and bacteria at 0.1 O.D. at 600 nm. The NB served as a negative control, whereas the bacterial suspension without phage was used as a positive control. The kinetic assay was performed for 12 h at 37 °C using a Multiskan SkyHigh Microplate Spectrophotometer (Thermo Scientific™).

### Biofilm dispersion assay

The biofilm formation assay was performed as described by Necel et al. [26], with a few modifications. *E. coli*/SSBS/P/22/15, *E. coli*/SSBS/P/22/29, and *E. coli*/SSBS/P/22/32 were tested for biofilm dispersion. A culture mixture with an O.D. of 0.3 at 600 nm was added to the plate with the three surfaces, and the plate was incubated for 18 h. Without disrupting the biofilm, the supernatant was removed, and 1 ml of antibiotic and/or phage lysate was added to the corresponding wells and incubated at 37 °C for 6 h. Well, which consisted of biofilm formed by a host and not treated with phages and/or antibiotics, was used as a positive control. The biofilm was washed with 1x PBS and stained with 1 ml of crystal violet for 20 min at RT. The excess crystal violet stain in each well was removed by washing with 1x PBS, then treating with 30% glacial acetic acid, and the O.D. was measured at 570 nm.

### Statistical tools

For the statistical analysis, the data were subjected to the GraphPad Prism 8.0 program, which executed Student’s t-test and one-way ANOVA at a significance level of 5% (α = 0.05).

## Results

### Biofilm formation assay of *E. coli* isolates on different surfaces

**Fig. 1:**
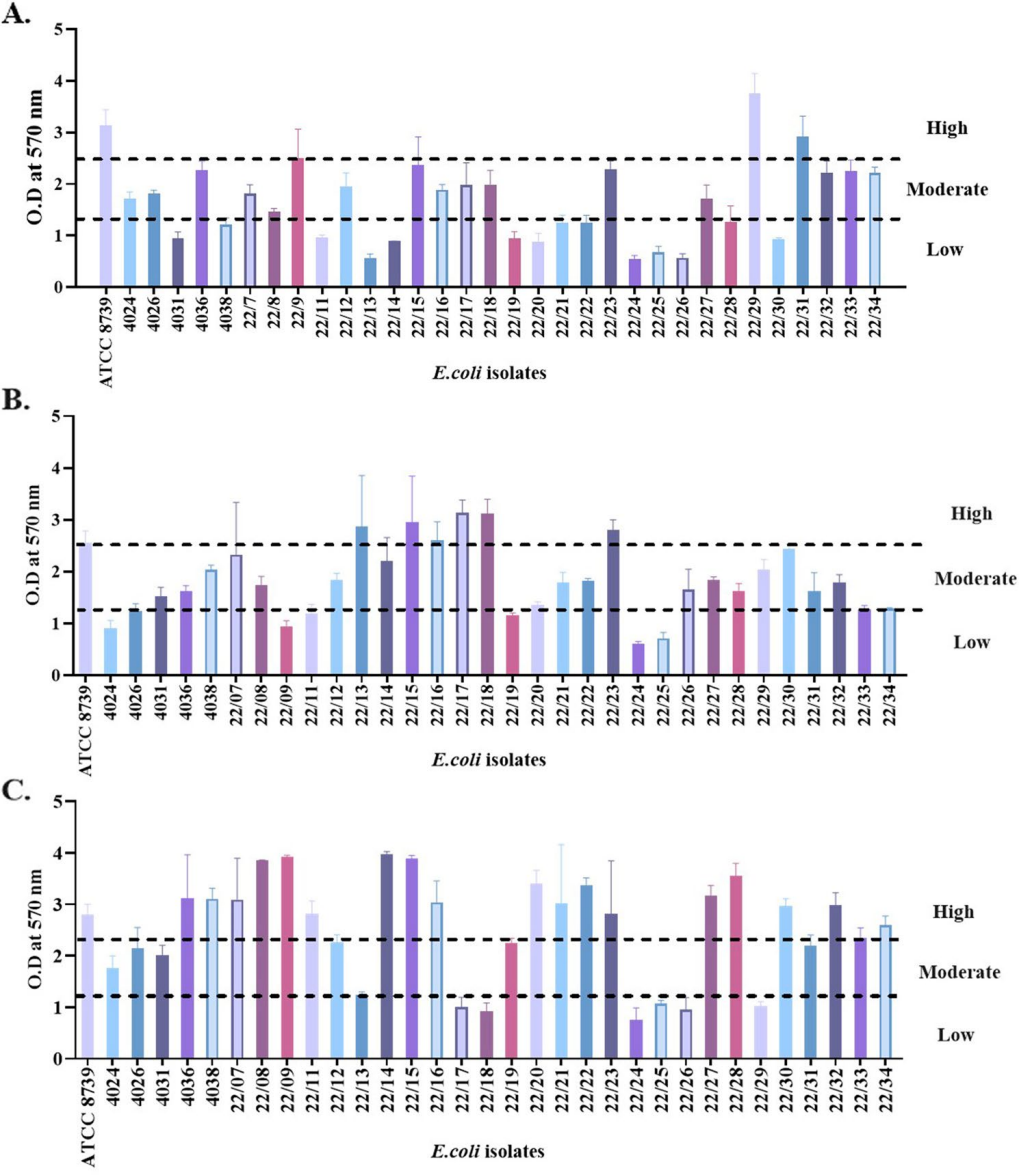
Biofilm formation by *E. coli* isolates on PP (**A**), SS (**B**), and PVC (**C**). The error bars represent the standard deviations from three replicate experiments

The biofilm-forming ability of 32 poultry *E. coli* isolates was examined via crystal violet staining of three surfaces: PP, SS, and PVC. These surfaces were chosen because they are regularly used in water-drinking vessels, housing/caging materials, and pipelines that supply water to poultry birds. PVC is the most susceptible of the three surfaces to APEC biofilm formation, followed by SS and PP. Isolates, namely, *E. coli*/SSBS/P/22/15, *E. coli*/SSBS/P/22/29 and *E. coli*/SSBS/P/22/32, were selected for further study from the 32 isolates, as they showed moderate or high biofilm production on all the surfaces. The classification of isolates as low, moderate, or high biofilm producers was performed as suggested by Guembe et al., wherein the cutoffs (based on tertile ranks) used to classify the strains according to biofilm production (CV) [29] and is represented in Fig. 1. The classification of the isolates into high, moderate, and low-biofilm producers is shown in Table 1.

**Table 1:** Number of isolates and their biofilm formation ability on PP, SS and PVC

Surface	High biofilm producers (%)	Moderate biofilm producers (%)	Low biofilm producers (%)
Polypropylene (PP)	25	43	31.25
Stainless Steel (SS)	21.87	62.5	15.6
Polyvinyl chloride (PVC)	62.5	25	18.75

### Minimum inhibitory concentration (MIC)

**Fig. 2:**
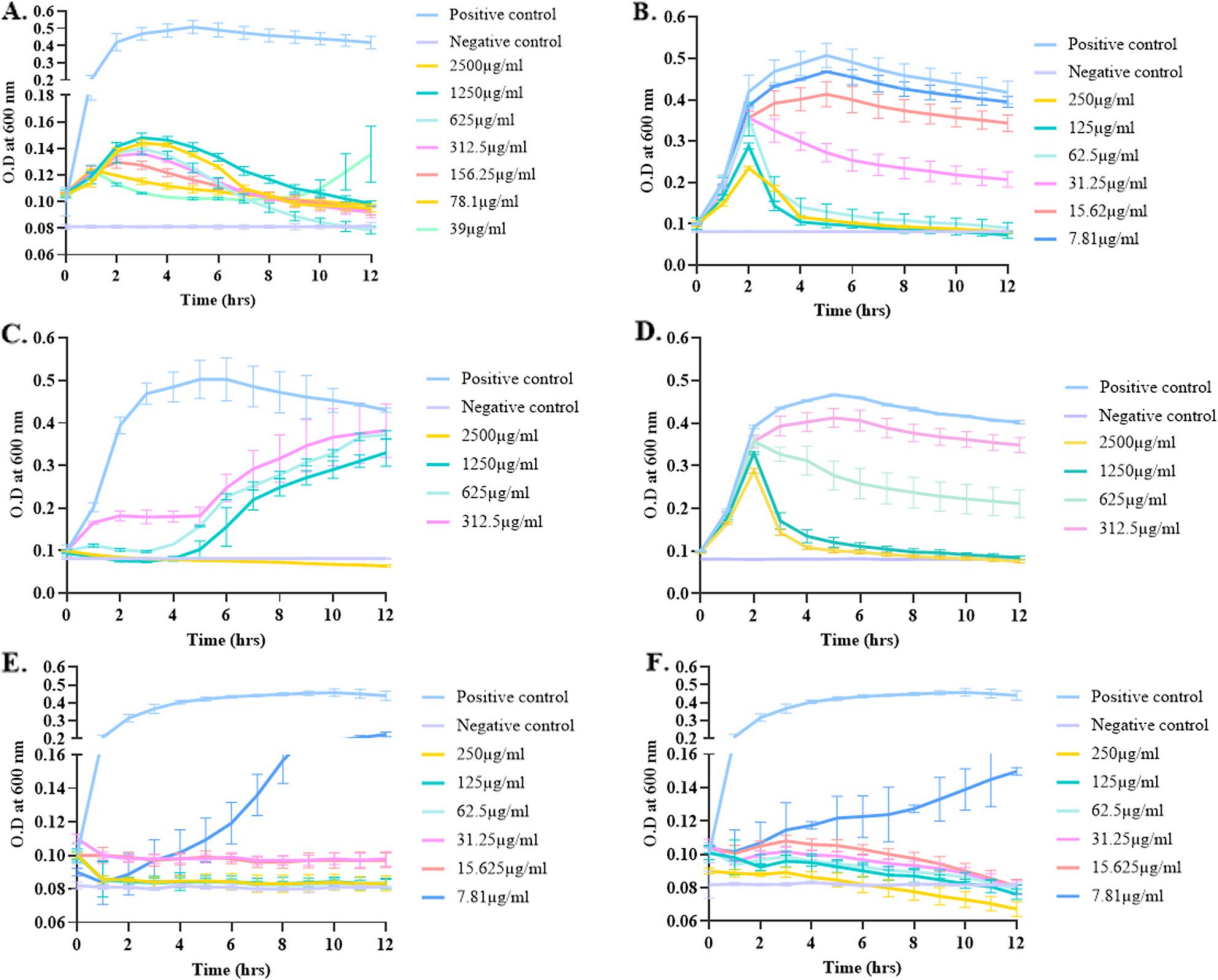
MIC determination assay of isolated *E. coli*/SSBS/P/22/15 planktonic cells with GEN (**A**) and CIP (**B**), *E. coli*/SSBS/P/22/29 planktonic cells with GEN (**C**) and CIP (**D**), and *E. coli*/SSBS/P/22/32 planktonic cells with GEN (**E**) and CIP (**F**). Host bacteria without antibiotics were considered positive controls, and NB served as the negative control. The error bars represent the standard deviations from three replicate experiments

The minimum inhibitory concentration was determined via the microdilution method for gentamicin (GEN) and ciprofloxacin (CIP), with concentrations ranging from 2500 µg/mL to 2.5 µg/mL. The MIC values of GEN for the *E. coli*/SSBS/P/22/15 and *E. coli*/SSBS/P/22/29 isolates were 78.1 µg/mL and 2500 µg/mL, respectively. Whereas MIC values of CIP for the *E. coli*/SSBS/P/22/15 and *E. coli*/SSBS/P/22/29 isolates were 31.25 µg/mL and 625 µg/mL, respectively. The MICs of these isolates against CIP were relatively lower than those against GEN. The MIC values for the *E. coli*/SSBS/P/22/32 isolates were the same for both antibiotics (GEN and CIP), i.e., 31.25 µg/mL. However, *E. coli*/SSBS/P/22/32 requires approx. 80-fold higher concentration to have a bactericidal effect (MBC of 2500 µg/mL) (Table 2). The observations revealed varying levels of antibiotic resistance among the isolates. The isolate exhibited comparatively lower resistance to gentamicin than to ciprofloxacin, although higher concentrations are needed for bactericidal effects. The isolate *E. coli*/SSBS/P/22/29 is highly resistant to both antibiotics, indicated with high MIC and MBC values. In contrast, the isolate *E. coli*/SSBS/P/22/32 is more sensitive to GEN as compared to CIP for bactericidal action. This highlights differences in antibiotic efficacy in the isolates, and the same is represented in Fig. 2.

**Table 2:** Minimum inhibitory concentration (MIC) and minimum bactericidal concentration (MBC) of the three isolates against the GEN and CIP antibiotics

Isolate	MIC GEN (µg/mL)	MBC GEN (µg/mL)	MIC CIP (µg/mL)	MBC CIP (µg/mL)
*E. coli*/SSBS/P/22/15	78.1	2500	31.25	250
*E. coli*/SSBS/P/22/29	2500	2500	625	2500
*E. coli*/SSBS/P/22/32	31.25	250	31.25	2500

*GEN* Gentamicin, *CIP* Ciprofloxacin

### Bacterial killing assay

**Fig. 3:**
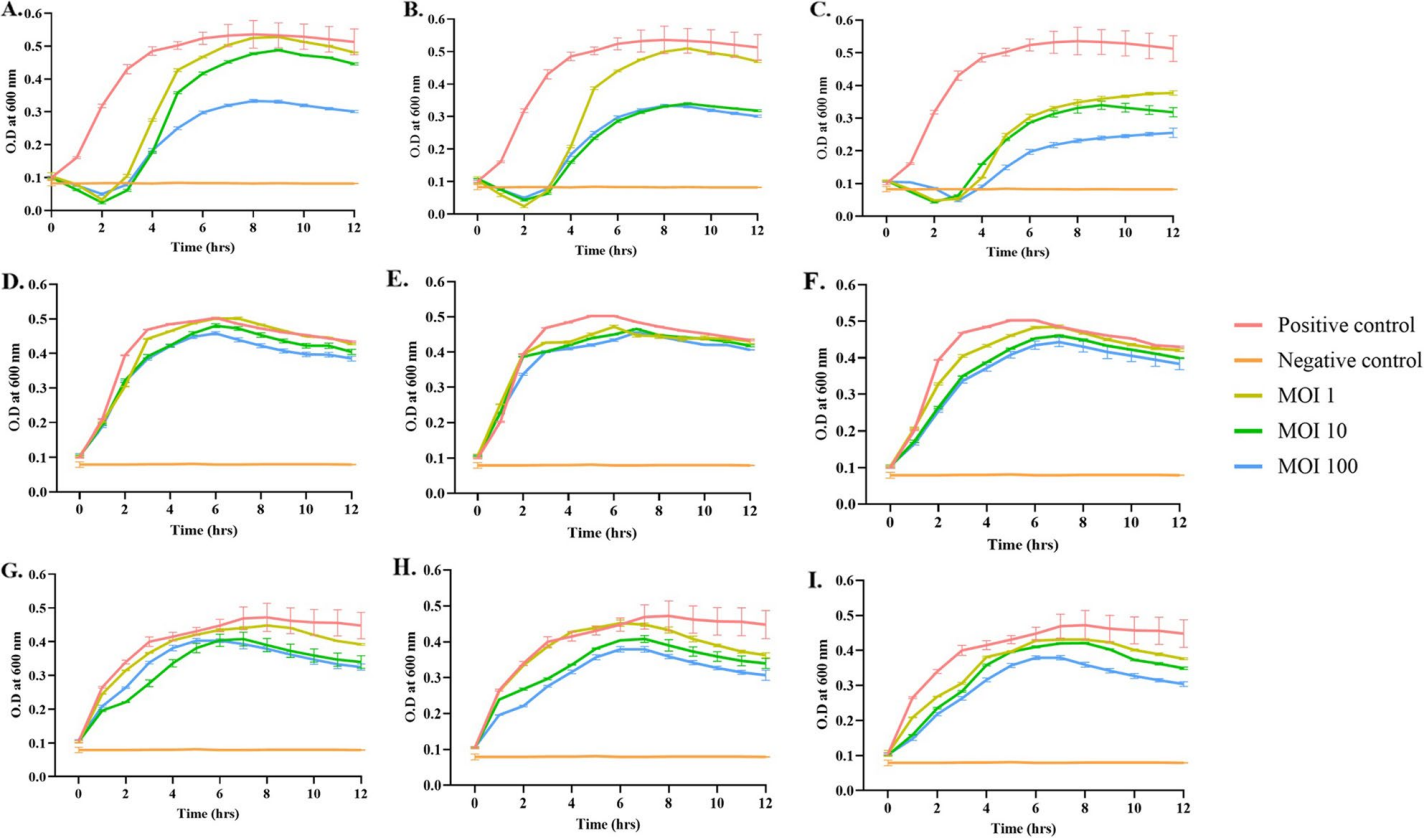
Host killing assay of *E. coli*/SSBS/P/22/15, *E. coli*/SSBS/P/22/29, and *E. coli*/SSBS/P/22/32 by EscoΦB-01 (**A**, **D**, **G**), EscoΦA-06 (**B**, **E**, **H**), and EscoΦA-07 (**C**, **F**, **I**), respectively. A host incubated without phage was used as a positive control, and NB was used as a negative control. The error bars represent the standard deviations of three replicate experiments

The microdilution plate method was used to determine the efficacy of the three phages; EscoΦB-01, EscoΦA-06 and EscoΦA-07. Phages were active against the strains at an MOI of 100, as shown in Fig. 3. Lysis efficiency of the phages was compared at 12th hour of incubation, where phage EscoΦB-01 showed the maximum lysis percentage of 41.6% at MOI 100 as compared to 10 and 13% at MOI 1 and MOI 10 for *E. coli*/SSBS/P/22/15 isolate. When tested with EscoΦA-06, MOIs of 10 and 100 resulted in similar lysis patterns (38% and 41%, respectively) In the case of EscoΦA-07, the lysis efficiency increased as MOI increased; The lysis of 26.5%, 38% and 50% was observed in MOI 1, MOI 10, and MOI 100. *E. coli*/SSBS/P/22/29, showed minimal lysis with all three phages. EscoΦB-01 resulted in 12.2%, EscoΦA-06 resulted in 7.1%, and EscoΦB-01 resulted in 10.9% lysis at a MOI of 100. When dealing with *E. coli*/SSBS/P/22/32, EscoΦB-01 showed the best lysis as compared to EscoΦA-06 and EscoΦA-07. These results reflect that the lysis pattern of the same phage differs in every host, even though they belong to the same species, and not only the phage and its dosage itself.

### Phage‒antibiotic synergism assay

**Fig. 4:**
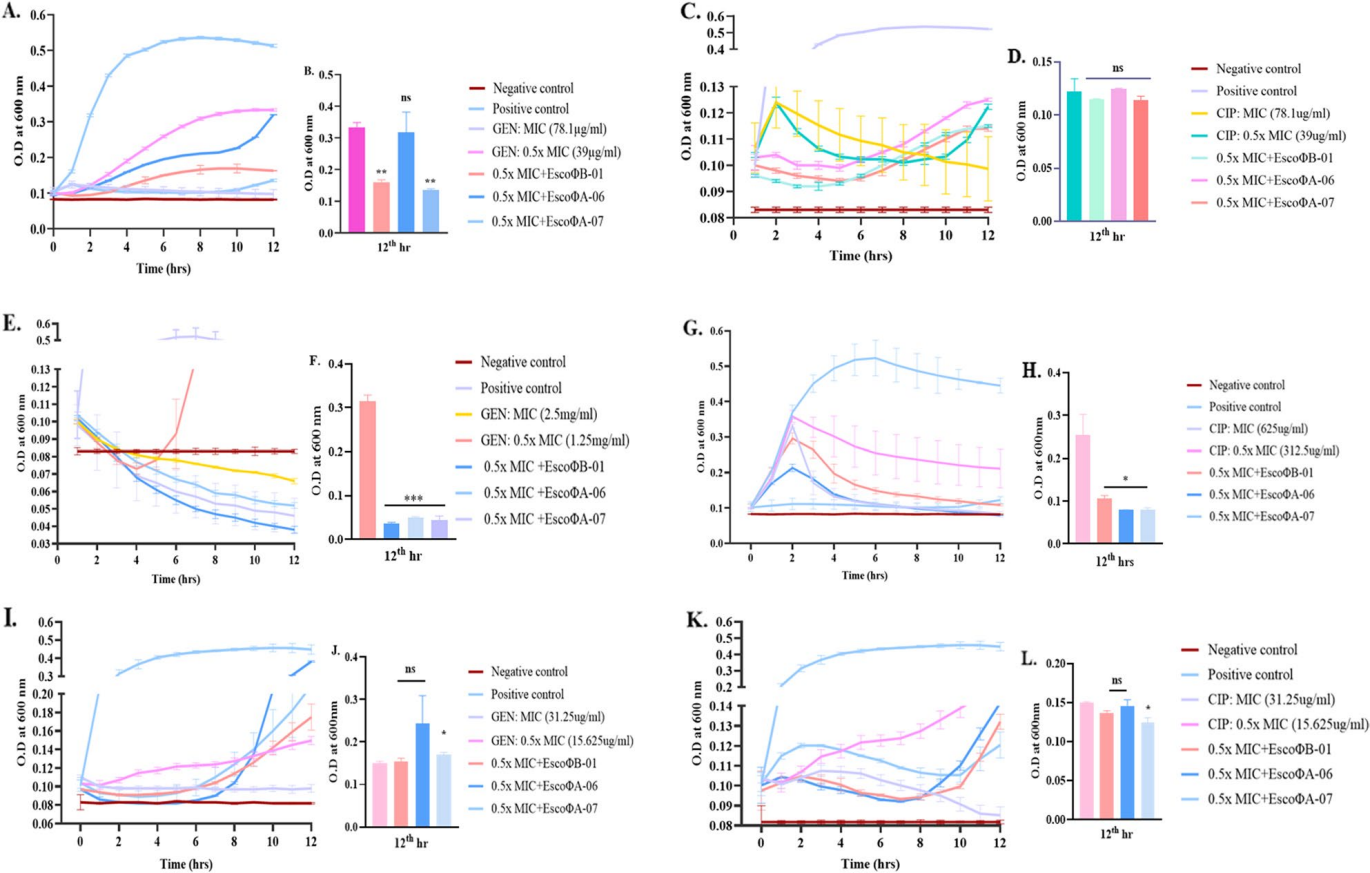
PAS assessment of *E. coli/SSBS/P/*22/15, *E. coli*/SSBS/P/22/29, and *E. coli*/SSBS/P/22/32 with GEN and phages; EscoΦB-01, EscoΦA-06, and EscoΦA-07 at an MOI of 100 (**A**, **E**, **I**) and CIP along with phages; EscoΦB-01, EscoΦA-06, and EscoΦA-07 at an MOI of 100 (**C**, **G**, **K**). Bar graphs represent the PAS of GEN with phages (**B**, **F**, **J**) and CIP with phages (**D**, **H**, **L**) at the 12th hour of the experiment. A host incubated without phage and/or antibiotic was considered a positive control, and NB was considered a negative control. The error bars represent the standard deviations of three replicate experiments

Bacteriophages and antibiotics were used together to assess lysis efficiency via the PAS treatment approach. When GEN at a sub-MIC (39 µg/mL) along with all three bacteriophages was tested for *E. coli*/SSBS/P/22/15, all the tested combinations resulted in reduced bacterial growth compared with the control, but the combination of sub-MIC concentrations and EscoΦB-01 significantly reduced bacterial growth. For *E. coli*/SSBS/P/22/29, complete bacterial inhibition was achieved within 12 h when sub-MIC (1.25 mg/mL) concentrations of all three phage combinations were used. *E. coli*/SSBS/P/22/32 exhibited total bacterial inhibition within 8 h at sub-MIC (15.625 µg/mL) concentrations, with the three phages depicted in Fig. 4.

### Biofilm dispersion

**Fig. 5:**
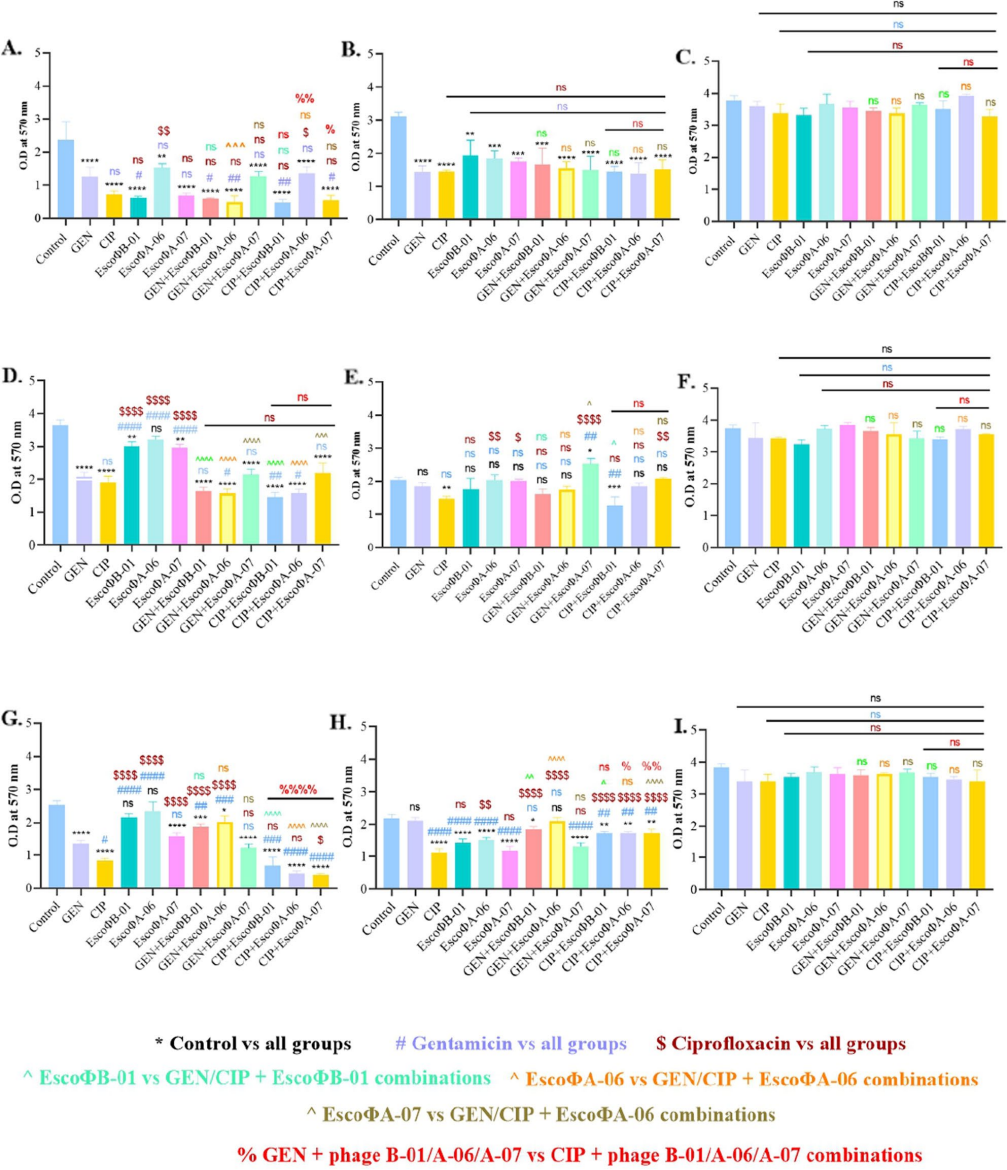
Biofilm dispersion of *E. coli/SSBS/P/*22/15, *E. coli*/SSBS/P/22/29, and *E. coli*/SSBS/P/22/32 by GEN and/or CIP and/or EscoΦB-01, EscoΦA-06 and EscoΦA-07 on different surfaces, including PP (**A**, **D**, **G**), SS (**B**, **E**, **H**), and PVC (**C**, **F**, **I**). A biofilm formed by a host without phages and/or antibiotics was used as a positive control. The error bars represent the standard deviation from triplicate experiments. Statistical significance was assessed using a one-way ANOVA at a significance level of 5% (α = 0.05)

The isolate *E. coli*/SSBS/P/22/15 had formed moderate biofilm on the PP surface, which was effectively dispersed by all the independent treatments of antibiotics and phages, whereas the most significant PAS was found with the combined treatment of EscoΦA-06 + GEN, when compared with the dispersion mediated independently by EscoΦA-06 and GEN on PP (Fig. 5A). Isolate *E. coli*/SSBS/P/22/15 showed high biofilm-forming ability on SS and PVC surfaces; thus, only independent treatments could effectively disperse biofilm on SS (Fig. 5B), while none of the combinations or independent treatment approaches could lyse biofilm formed on PVC (Fig. 5C).

Although the isolate *E. coli*/SSBS/P/22/29 demonstrated high biofilm-forming ability on the PP surface, both the antibiotic and phage combination tests showed significant biofilm dispersion. A combination of EscoΦB-01 + CIP showed the most effective dispersion not only on PP (Fig. 5D**)** but also on the SS surface, where none of the other combinations could mediate a significant reduction (Fig. 5E). Even though the isolate *E. coli*/SSBS/P/22/29 had low biofilm-forming ability on PVC, none of the approaches could disperse the biofilm (Fig. 5F).

The isolate *E. coli*/SSBS/P/22/32 formed a moderate biofilm on the PP surface, and all treatment approaches showed effective reductions in biofilm. Although GEN + phage groups also exhibited PAS, when compared with CIP + phages, CIP + phages were more effective, and the combination of EscoΦA-07 + CIP had the most significant dispersion of the biofilm (Fig. 5G). Surprisingly, except for GEN, the other approaches reduced biofilm formation on the SS surface, but all combination tests showed that they mediated PAA and could not disperse the biofilm effectively (Fig. 5H). As with other isolates, neither treatment approach reduced biofilm formed by *E. coli*/SSBS/P/22/32 on the PVC surface (Fig. 5I).

## Discussion

The present study evaluated whether avian pathogenic *E. coli* preferentially adheres to surfaces commonly used in the poultry industry, such as PP, SS, and PVC. The in vitro bacterial-killing activity of the three phages against selected APEC isolates was assessed alone and in combination with antibiotics. The effects of combinations of antibiotics, bacteriophages, and phage-antibiotic combinations on biofilms formed on poultry-related surfaces by APEC isolates were assessed. It was found that PAS helps in the effective elimination of biofilms.

Colibacillosis is a serious problem in the poultry industry, caused by APEC infections that can occur sporadically and impose financial burdens [30]. In addition, there is no effective vaccine to treat these infections, and antibiotics are less effective, as bacteria are resistant to commonly used antibiotics in poultry [31]. The reason for infections may be the conditions in which birds are housed, which provide bacteria with ideal conditions for adherence, multiplication, and biofilm formation. Biofilms are also harmful to humans because they facilitate the exchange of antimicrobial resistance genes [32], virulence genes *that allow APEC to survive in host serum* [33], *and hylfF*,* which is found in* many other *E. coli* strains that cause human infections [25, 34].

The surfaces used in the study are mostly housing materials, including stainless-steel cages, water pipes for transporting water, and PVC and PP poultry feeders and drinkers. Biofilm formation on PVC was the highest at 82%, followed by SS at 79% and PP at 64%. Strong biofilms were primarily observed on PVC. The percentages of biofilms on SS and PP were moderate at 58.8% and 41%, respectively. Biofilm formation depends on a range of biotic and abiotic factors. Biotic factors, such as the adhesive genes of APEC, such as *FimH*, help bacteria mediate adhesion and form biofilms [2]. Abiotic factors, such as surface hydrophobicity, surface charge, and roughness, promote bacterial adhesion. PVC is hydrophobic and has a rough surface that may promote bacterial adhesion [35]. SS is often regarded as an antimicrobial surface and was also shown to form bacterial biofilms in this study. The SS used in this study is SS 304, an affordable SS containing 18–20% chromium and 8 − 2% nickel, commonly used in poultry farms and many small businesses. In support of our observation, an earlier investigation carried out by Tran et al. revealed that SS consisting of nickel can attract bacteria and promote bacterial adhesion, although chromium, on the other hand, shows better antibacterial properties mediated by the material itself and supports phage-mediated lysis, representing a differential bacterial response to the surfaces [36, 37]. The higher-grade SS used in heavy industries has premium elements, such as molybdenum and titanium oxide, as coating additives, which reduce bacterial adhesion [38]. However, these materials are not used in the poultry industry because of the high cost of premium-grade steel. Therefore, it is important for these industries to follow thorough cleaning and sanitation procedures to minimise the risk of bacterial contamination.

PP is a crucial plastic in the material industry and is also used in the poultry industry. The results revealed that, compared with the other two surfaces, the PP surfaces support less biofilm formation. According to the parameters discussed by Zheng et al., the same surface shows that hydrophobicity and roughness play important roles [35]. Compared with PVC and SS 304, PP has a smoother surface with less surface roughness. As a result, bacteria cannot adhere to PP and form robust biofilms as they can on the other two surfaces [39]. This feature makes PP the preferred material for surfaces in biofilm-prone environments, such as food processing or medical settings, reduces the risk of bacterial contamination, and ensures a cleaner, safer environment for products and personnel. The findings of this study suggest that the material composition and surface characteristics are important parameters for selecting appropriate materials.

Three of the 32 isolates, *E. coli*/SSBS/P/22/15, *E. coli*/SSBS/P/22/29, and *E. coli*/SSBS/P/22/32, were selected for further analysis based on their high/moderate biofilm formation ability on different surfaces and susceptibility to phages. Isolate *E. coli*/SSBS/P/22/15 formed moderate Biofilm on PP, whereas it formed high biofilm on SS and PVC. Isolate *E. coli*/SSBS/P/22/29 formed moderate biofilm on SS, whereas it was high on PP and PVC. While isolate *E. coli*/SSBS/P/22/32 formed moderate biofilm on PP and SS, but high biofilm on PVC. These isolates represent differential biofilm formation on different surfaces, along with differential lysis mediated by phages. EscoΦA-06, EscoΦA-07, and EscoΦB-01 caused bacterial lysis of *E. coli*/SSBS/P/22/15 and *E. coli*/SSBS/P/22/32. However, *E. coli*/SSBS/P/22/29 showed the least lysis by all these phages and was selected for the study to analyse the dynamics of this isolate in poultry settings. Lysis of isolates reveals information about phage specificity and whether phage‒antibiotic synergy affects it. No phage could lyse all bacterial isolates when UPEC is treated with bacteriophages [40].

The antibiotics CIP and GEN, which belong to the fluoroquinolone and aminoglycoside classes, with distinct modes of action, were selected for the study because they are widely used in the poultry industry to treat bacterial infections and are considered sentinel antibiotics, serving as proxy indicators of MDR. The antibacterial action of CIP is mediated by inhibiting bacterial DNA gyrase and topoisomerase IV, thereby preventing DNA replication. Whereas, GEN mediates its antibacterial effect by inhibiting protein synthesis and disrupting cell membrane integrity. Thus, when these antibiotics are used with phages for a combinational treatment approach, the combined antibacterial effect could be either synergistic or antagonistic due to the varying mechanisms of action induced. It has been observed by Morris et al. that the combined effect of PAW33 phage with CIP exhibited synergy for all three targeted *P. aeruginosa* strains [41]. A recent study has showcased that temperate phages could also be used as synergizing adjuvants with CIP, assessed for *Burkholderia cepacia complex* (Bcc) species, expanding the sustainability of phage therapies by leveraging tPAS (Temperate phage-antibiotic synergy) as a tool to combat pathogens [42].

In an earlier study, Koratkar et al. [25], the resistance to CIP and GEN was found to be 100% and 91.3%, respectively. The GEN-MIC values for *E. coli*/SSBS/P/22/15, *E. coli*/SSBS/P/22/29, and *E. coli*/SSBS/P/22/32 were 31.25 µg/mL to 2500 µg/mL, which are relatively higher than the normal range. This high MIC could be attributed to antibiotic misuse in the poultry industry, which contributes to resistance. On the other hand, compared with GEN, CIP has a lower MIC, ranging from 31.25 µg/mL to 125 µg/mL, possibly because it is used less frequently in the sector.

The three phages were evaluated for their in vitro bactericidal activity, and the efficacy of phage combinations with antibiotics was subsequently tested in planktonic cells. The concentrations of antibiotics used were the MIC and 0.5x MIC (referred to as sub-MIC), combined with a MOI of 100 for all three phages. MOI 100 was used for combination assessments, as lower MOIs did not result in effective lysis. Almost all the combinations significantly reduced growth. The *E. coli*/SSBS/P/22/32 isolate, when combined with gentamicin and EscoΦA-06, showed a late, rapid increase in growth after 9 h of incubation, similar to the positive control at the end. The combination assay demonstrated how these antimicrobials interact with bacteria together under planktonic conditions and provided insight into their possible effects on biofilms.

The biofilms of these isolates on all three surfaces were subjected to biofilm dispersion in the presence of antibiotics, phages, or their respective combinations at various sub-MIC antibiotic concentrations and an MOI 100 concentration of phages. PP showed the greatest dispersion among the three surfaces and was treated individually with phages and in combination with antibiotics. Lower surface roughness and overall smoothness may prevent biofilm retention on the surface for longer periods, allowing antimicrobial agents to interact more readily with weakly attached biofilms [43]. Garcia et al. reported that Salmonella bacteria grown on glass surfaces were more susceptible to phage dispersion and suggested that the glass material interferes with biofilm formation [44]. In most of the combinations with the *E. coli*/SSBS/P/22/15 and *E. coli*/SSBS/P/22/32 isolates, the dispersion was more than 50% greater than that of *E. coli*/SSBS/P/22/29. The primary reason may be the greater lytic ability of the phages against the isolates, resulting in greater biofilm dispersion.

Biofilm dispersion on SS results in a very different outlook. The *E. coli*/SSBS/P/22/15 isolate showed the greatest biofilm dispersion, possibly due to increased phage lytic activity. All three isolates showed efficient synergistic activity. However, PAS with CIP had antagonistic effects on *E. coli*/SSBS/P/22/29 and *E. coli*/SSBS/P/22/32. In an ideal scenario, the combination should have the same or more dispersion than either of its counterparts. One reason may be that antibiotics and phages interfere with each other’s mechanisms of action, so they do not have bactericidal effects when used in combination. Gu Liu et al. reported similar findings when CIP was used at relatively high doses in combination with bacteriophages to degrade planktonic cells [45]. Processes important for phage diffusion and replication can be restricted, as biofilms harbor strong EPS, and phages may be unable to replicate well due to inhibition of DNA gyrase, which disrupts the replication cycle and protein synthesis [46].

The PVC material, which has the highest molecular roughness and hydrophobicity among the three materials, allows the least biofilm dispersion. This is concerning not only because biofilms are not eradicated, but also because biofilm formation is most efficient. A study performed by Zhao et al. also suggested that biofilms formed on PVC surfaces are the most complex and have a multicellular structure [47]. Novel strategies to combat PVC biofilms must be developed, given the challenges they pose in poultry settings. One possible solution is the use of regular cleaning and disinfection measures specifically suited to PVC surfaces in poultry settings [48]. Additionally, exploring alternative materials with stronger antibacterial properties could help mitigate biofilm formation on PVC surfaces.

The results obtained in this study indicate that phage antibiotic synergism is a positive way to combat biofilms and treat MDR infections. However, in a particular situation, phage‒antibiotic antagonism is also observed. While this study addresses in vitro biofilm degradation, PAS can also help eliminate or reduce biofilms in chickens and turkeys in vivo [49]. While phages are a potential alternative for treating AMR infections, not all bacteriophages can lyse all bacterial isolates due to their restricted host range [50, 51]. One possible solution to this situation is the use of a phage cocktail [52]. To prevent phage‒antibiotic antagonism, the choice of antibiotics, their concentration, and phages must be made judiciously to prevent further antibiotic resistance that can spread rapidly in poultry settings [53]. In situations where the development of new antibiotics is scarce, repurposing available antibiotics with greater caution is a wise option [25, 54]. With increasing antibiotic resistance, many of these new phages should be screened for their antimicrobial activity against APEC to prevent the spread of AMR in poultry settings. In addition, new methods should be developed to use phages in real-world settings to understand their interactions outside the laboratory.

## Conclusion

This study highlights the challenges associated with APEC infections in the poultry industry, particularly biofilm formation on commonly used surfaces and antibiotic resistance. The study revealed that biofilm formation varies significantly by surface material, with PVC resulting in the greatest biofilm formation, followed by SS and PP. The bacteriophages EscoΦA-06, EscoΦA-07, and EscoΦB-01 showed promising in vitro antibacterial activity against APEC isolates. The study also revealed the complexity of phage‒antibiotic interactions, with some combinations enhancing biofilm dispersion and bacterial killing, while others showed antagonistic effects, especially with CIP. This study revealed that PAS has potential as an effective strategy for controlling biofilm-associated infections in poultry. However, further research is needed to optimise phage‒antibiotic combinations and explore their practical applications under field conditions. Additionally, stringent biosecurity measures, including regular cleaning and sanitisation, should be implemented to minimise biofilm formation on materials such as PVC, SS, and PP. Integrating phage therapy into poultry health management could serve as a sustainable approach to reduce the use of conventional antibiotics and combat the spread of AMR.

## Data Availability

All data generated or analysed during this study are included in this published article.

## Abbreviations

APEC: Avian pathogenic Escherichia coli
CIP: Ciprofloxacin
ESBL: Extended-spectrumβ-lactamases
ExPEC: Extraintestinal pathogenic E. coli
GEN: Gentamicin
MBC: Minimum bactericidal concentration
MDR: Multidrug resistance
MIC: Minimum inhibitory concentration
NB: Nutrient broth
NMEC: Neonatal meningitis E. coli
PAA: Phage‒antibiotic antagonism
PAS: Phage‒antibiotic synergy
PP: Polypropylene
PVC: Polyvinyl chloride
SEPEC: Sepsis-associated E. coli
SS: Stainless steel
UPEC: Uropathogenic E. coli
